# Publications on cross-cultural aspects of eating disorders

**DOI:** 10.1186/2050-2974-1-4

**Published:** 2013-01-22

**Authors:** Nerissa Li-Wey Soh, Garry Walter

**Affiliations:** 1 Child and Adolescent Mental Health Services, Northern Sydney Local Health District, Coral Tree Family Service, PO Box 142, North Ryde, NSW 1670, Australia; 2 Discipline of Psychiatry, University of Sydney, and Child and Adolescent Mental Health Services, Northern Sydney Local Health District, Coral Tree Family Service, PO Box 142, North Ryde, NSW 1670, Australia

**Keywords:** Cross-cultural, Eating disorders, Bibliometric, African, Middle-Eastern, South European, Asian, Pacific Islander, Hispanic

## Abstract

**Background:**

The prevalence of eating disorders in the non-Western world appears to be increasing and much research into the cross-cultural aspects of eating disorders is needed. This bibliometric study analyses the profile of cross-cultural studies into eating disorders published from 1970 through to 2011.

**Results:**

1,417 articles were indexed by Medline and PsychInfo from 1970 to 2011. There has been an exponential increase in publications in this field. Four articles were published in 1970–74 and this increased to 427 in 2004–9. Comparative and empirical studies were the most common types of publications. Of all the ethnic groups studied, Africans and African Americans were subject of the most publications. Pacific Islanders and South Europeans had the fewest publications.

**Conclusion:**

It is heartening that there has been a large increase in published studies about eating disorders across cultures. This suggests greater awareness and interest in the field. However, the results from one particular ethnic group cannot always be applied directly to another. Some ethnic and cultural groups have been poorly studied and warrant more research attention. As more patients from such backgrounds present for treatment, more research is needed to provide culturally appropriate and acceptable care.

## Background

Eating disorders were previously thought to be “Western culture-bound syndromes”, with non-Western individuals being considered immune. Articles on non-Western populations and eating disorders first appeared in the international scientific literature in the 1970s [1]. The prevalence of eating disorders in non-Western groups has been found to be generally lower than that in Western populations but appears to be increasing [1-3]. This epidemiological pattern may not reflect true incidence rates but instead may indicate improving international awareness and identification. For example, anorexia nervosa was already documented in Japan and in Japanese in 1941 and Japanese records detail a psychological “non-eating” illness dating back to the 17^th^-18^th^ century [1]. International published research in recent years also shows mixed conclusions in relation to whether eating disorders present differently in different cultural groups [1]. Debate over the criteria for diagnosing eating disorders in non-Western, non-Caucasian groups [4], together with the frequent failure of health professionals themselves to identify eating disorders in these populations [5], suggests that the prevalence in such groups may currently be underestimated.

Despite the apparent increase of eating disorders in non-Western groups [1-3], there has been relatively little research published on eating disorders across cultures. Studies of eating disorders in non-Western cultural groups and countries have focused mainly on African Americans and results from such research cannot necessarily be applied to other non-Western populations. More information is needed to provide culturally appropriate and acceptable treatments for individuals with eating disorders from diverse cultural backgrounds.

Recent years have also witnessed an increasing popularity in bibliometric studies, which analyse publication patterns provide information about the current status and research priorities of particular scientific and medical fields [6-8]. To date, bibliometric research regarding articles published about the various ethnic and cultural dimensions of eating disorders has not been undertaken. The present study aims to provide a profile of the articles about eating disorders across cultures which were published from 1970 through to 2011.

## Methods

Medline and PsycInfo were selected as the databases, as they are extensively used by health researchers and clinicians. Citations relating to eating disorders and ethnic and cultural groups were then retrieved (search terms and strategies are available in the Additional file 1: Appendix).

Following retrieval of all citations, duplicates were removed with the preference of retaining Medline citations. Citations were then manually culled to remove articles not directly related to eating disorders as a psychiatric illness and articles not related to cross-cultural issues. Citations were retained if they were: on non-Western study samples, including if they were in non-Western languages; on ethnic minority groups in a Western country; solely on Western populations but which studied two or more countries and compared cultures; validated psychometric tools in non-English languages, including “Western European” languages such as French or the Scandinavian languages; on body dysmorphia, body image related to body building, or muscle dysmorphia; journal articles reviewing books; editorials, letters or comments. Citations were excluded if they: focused on obesity and did not include a significant body image and psychopathological component; were about pica, geophagia or infant feeding difficulties; focused on socio-economic status and socio-cultural issues and did not have a significant ethnicity or cross-country component; were on body image or body perception following surgery, cancer, burns and amputations but did not have an eating disorder psychopathological component; focused on eating habits or patterns without a psychological component; focused on the colour of skin or hair without a body shape or weight factor; were on sexual orientation but without a significant ethnicity or cross-country component; were solely on Western populations and cultures, including USA, Australia, New Zealand, United Kingdom, Canada, France, Germany and Scandinavia; did not address ethnicity or culture even though the article was in a non-English language; or were for grey literature (e.g., dissertation abstracts, books, book chapters or non-peer reviewed articles).

Culling was undertaken using titles, abstracts and key words. If the citation was still ambiguous at this point, the entire article was sourced to determine whether it should be included. The final set of citations was then broken down by five-year intervals of publication: 1970–74, 1975–79, 1980–84, 1985–89, 1990–94, 1995–99, 2000–4 and 2005–9. Articles published in 2010–11 formed a final set. Each set was then analysed by ethnic/cultural group and by publication or study type. Citations were also categorized by language of publication, that is, English versus non-English.

## Results

There were 1,417 citations which met the inclusion criteria. Table 1 shows the number of citations in each set by language and by ethnic group.

**Table 1:** Number of citations per five-year interval, by language and ethnic group

	**1970-74**	**1975-79**	**1980-84**	**1985-89**	**1990-94**	**1995-99**	**2000-4**	**2005-9**	**2010-11**	**Total (1970-2011)**
Number of citations	4	7	14	62	120	212	352	427	219	1417
Language (% citations in time interval)										
English	3 (75)	3 (43)	13 (93)	59 (95)	115 (96)	203 (96)	305 (87)	374 (88)	191 (87)	1266 (89)
Non-English	1 (25)	4 (57)	1 (7)	3 (5)	5 (4)	9 (4)	47 (13)	53 (12)	28 (12)	151 (11)
Ethnic group (% citations in time interval)										
African	1 (25)	1 (14)	8 (57)	187 (29)	38 (32)	71 (33)	98 (28)	75 (17)	36 (16)	346 (24)
Hispanic or Latin American	0 (0)	0 (0)	0 (0)	6 (10)	5 (4)	12 (6)	23 (7)	58 (14)	37 (17)	141 (10)
East European	0 (0)	0 (0)	0 (0)	0 (0)	4 (3)	5 (2)	14 (4)	29 (7)	10 (5)	62 (4)
South European	0 (0)	1 (14)	0 (0)	0 (0)	2 (2)	5 (2)	0 (0)	0 (0)	1 (0.5)	9 (0.6)
South Asian	0 (0)	0 (0)	0 (0)	1 (2)	4 (3)	5 (1)	5 (1)	5 (1)	6 (3)	26 (2)
East and South-East Asian	0 (0)	0 (0)	3 (21)	2 (3)	14 (12)	13 (6)	13 (4)	15 (4)	13 (6)	73 (5)
Pacific Islands and Oceania	0 (0)	0 (0)	0 (0)	0 (0)	1 (0.8)	4 (2)	2 (0.6)	1 (0.2)	3 (1)	11 (0.8)
Middle East	1 (25)	1 (14)	0 (0)	1 (2)	9 (8)	5 (2)	12 (3)	20 (5)	10 (5)	59 (4)

Citations by publication type are shown in Table 2.

**Table 2:** Number of citations by type of publication (% citations in time interval)

	**1970-74**	**1975-79**	**1980-84**	**1985-89**	**1990-94**	**1995-99**	**2000-4**	**2005-9**	**2010-11**	**Total (1970-2011)**
Number of citations	4	7	14	62	120	212	352	427	219	1417
Reviews	0 (0)	2 (29)	1 (7)	3 (5)	10 (8)	19 (9)	20 (6)	11 (3)	5 (2)	71 (5)
Case studies	0 (0)	0 (0)	0 (0)	10 (16)	10 (8)	9 (4)	12 (3)	12 (3)	3 (1)	31 (2)
Validation studies	0 (0)	0 (0)	0 (0)	0 (0)	0 (0)	0 (0)	5 (1)	14 (3)	12 (5)	31 (2)
Editorials	0 (0)	0 (0)	0 (0)	0 (0)	1 (0.8)	2 (0.9)	4 (1)	3 (0.7)	0 (0)	10 (0.7)
Comparative studies*	0 (0)	0 (0)	0 (0)	11 (18)	39 (33)	75 (35)	78 (22)	92 (22)	35 (16)	330 (23)
Randomized controlled trial	0 (0)	0 (0)	0 (0)	0 (0)	2 (2)	2 (0.9)	1 (0.3)	7 (2)	2 (0.9)	14 (1)
Empirical study**	0 (0)	0 (0)	0 (0)	46 (74)	65 (54)	132 (62)	235 (67)	275 (64)	140 (64)	346 (24)

* This category of publication is only available in Medline.

** This category of publication is only available in PsycInfo.

## Discussion

To our knowledge, this is the first study to assess the number of articles published on eating disorders across cultures. The number has increased exponentially between 1970 and 2011, indicating considerable and increasing interest and awareness in this area. The most recent two-year time bracket (2010–11) showed 219 articles had been published, nearly double that in the five-year bracket of 1990–94. This is heartening, given that from 1996 to 2010 alone, 4,162 articles were published by six specialized eating disorders journals [6].

Of all the cultural groups analysed, the African group (including African Americans) has been the best studied. This group was one of the first to be investigated, and currently one-fifth of cross-cultural articles about eating disorders have been on African cultural and ethnic groups. Interestingly, the proportion of publications on this group has decreased slightly over the last decade. However, studies of African groups still form a substantially greater proportion than do studies of all the other groups investigated. The second-most studied was the Latin American and Hispanic group. All the other ethnic groups have had very few articles published.

It is not clear why the South European group should have been so poorly studied, particularly considering Spain was included in this group and that the Latin American/Hispanic (and thus predominantly Spanish-speaking) group has been comparatively well represented. It may reflect a publication and indexing bias towards US articles, which in turn have a particular interest in both African American and Hispanic groups. The Pacific Islands and South Asia have also been poorly represented. The number of articles published on Middle Eastern and East European groups, while small, has steadily increased over time. For East and South-East Asians, the percentage of manuscripts devoted to them has plateaued. The limited epidemiological data suggests that the prevalence of eating disorders in non-Western groups is on the rise [2,9]; however, this may merely reflect growing awareness and identification of eating disorders in these populations. Nevertheless, it is concerning that there are so few studies of many of these groups and that the number and proportion of studies is not necessarily increasing over time.

With regard to the types of papers published in cross-cultural eating disorders research, most are empirical or comparative studies. It was expected that there would be few controlled trials, as such research is very difficult to undertake in eating disorders in general. In earlier decades, empirical studies formed the vast majority of cross-cultural publications and this remains the case in 2010–11, although there appears to be an increase in validation studies. This latter finding should be interpreted cautiously, as the number of publications classified as validation or psychometric studies is still small.

The limitations of this analysis are that we used Medline and PsycInfo, which are popular databases but mostly index journal articles which are in English or have English language abstracts [10,11]. This potentially makes non-English language research “invisible” to academics in English-language settings, who are thus not aware of and/or not accessing such material. Our analyses show a small but steady growth in the number of articles indexed by Medline and PsycInfo which are published in languages other than English. This may not be a true reflection of publication rates of non-English language articles, and non-English databases should be searched in future studies. For example, Chisuwa and O’Dea [3] have searched Japanese databases to locate eating disorder articles in Japanese journals, citing 11 papers, including publications on the prevalence of eating disorders in Japan, treatment infrastructure and body image. Another limitation to our study is that the subject headings used to extract citations from Medline and PsycInfo did not yield mutually exclusive sets of articles. For example, Egyptian populations were indexed as both Middle Eastern and African. Studies which cover more than one ethnic group would also be indexed and counted under individual ethnicgroups. Finally, some articles were indexed under more than one publication type.

## Conclusion

The increasing prevalence or identification of eating disorders in non-Western populations and the low numbers of research studies published in these population groups indicate a dire need to increase research in this field. Treatment rates are lower than in Western groups [3,9,12] and the dearth of information on eating disorders in non-Western groups is a probable barrier to their access to care [9]. Much more research is needed in this area to equip health professionals to remove barriers to access and deliver culturally acceptable and effective treatments to patients of non-Western backgrounds.

## Competing interests

The authors declare they have no competing interests.

## Authors’ contribution

NS contributed to study design, bibliometric data extraction and analyses, data interpretation and drafting of the manuscript. GW contributed to study design, data interpretation and drafting of the manuscript. Both authors read and approved the final manuscript.

## Supplementary Material

Additional file 1 **Appendix.**Table A: Initial Medline subject headings and search strategy for cross-cultural articles on eating disorders. Table B: Initial PsycInfo subject headings and search strategy for cross-cultural articles on eating disorders. Table C: Subject headings used for each ethnic group analysed. Table D: Limits applied for each publication type analysed. Click here for file
