# Supplementary material for: Publications on cross-cultural aspects of eating disorders
Source: J Eat Disord. 2013 Jan 22;1:4. doi: 10.1186/2050-2974-1-4 (PMC3776204; doi:10.1186/2050-2974-1-4)
Supplement: Additional file 1 — Appendix.Table A: Initial Medline subject headings and search strategy for cross-cultural articles on eating disorders. Table B: Initial PsycInfo subject headings and search strategy for cross-cultural articles on eating disorders. Table C: Subject headings used for each ethnic group analysed. Table D: Limits applied for each publication type analysed. [file 2050-2974-1-4-S1.doc]

(APPENDIX)

Table A: Initial Medline subject headings and search strategy for cross-cultural articles on eating disorders

| - exp eating disorders/ - exp anorexia nervosa/ - exp binge-eating disorder/ - exp bulimia nervosa/ - exp Bulimia/ - exp Body Image/ | and | - exp acculturation/ or exp cross-cultural comparison/ or exp cultural characteristics/ or exp cultural diversity/ or exp ethnology/ - africa/ or exp africa, northern/ or exp "africa south of the sahara"/ - exp African Americans/ - exp south america/ or exp argentina/ or exp bolivia/ or exp brazil/ or exp chile/ or exp colombia/ or exp ecuador/ or exp french guiana/ or exp guyana/ or exp paraguay/ or exp peru/ or exp suriname/ or exp uruguay/ or exp venezuela/ - exp central america/ or exp belize/ or exp costa rica/ or exp el salvador/ or exp guatemala/ or exp honduras/ or exp nicaragua/ or exp panama/ or exp mexico/ - americas/ or exp latin america/ - exp hispanic americans/ or exp mexican americans/ - europe/ or exp europe, eastern/ or exp albania/ or exp baltic states/ or exp estonia/ or exp latvia/ or exp lithuania/ or exp bosnia-herzegovina/ or exp bulgaria/ or exp "republic of belarus"/ or exp croatia/ or exp czech republic/ or exp hungary/ or exp "macedonia (republic)"/ or exp moldova/ or exp montenegro/ or exp poland/ or exp romania/ or exp russia/ or exp bashkiria/ or exp dagestan/ or exp moscow/ or exp siberia/ or exp serbia/ or exp slovakia/ or exp slovenia/ or exp ukraine/ or exp yugoslavia/ - exp bangladesh/ or exp india/ or exp sikkim/ or exp pakistan/ or exp sri lanka/ - Ethnic Groups/ - exp oceania/ or exp australasia/ or exp australia/ or exp new zealand/ or exp pacific islands/ or exp melanesia/ or exp micronesia/ or exp polynesia/ - exp middle east/ or exp afghanistan/ or exp bahrain/ or exp iran/ or exp iraq/ or exp israel/ or exp jordan/ or exp kuwait/ or exp lebanon/ or exp oman/ or exp qatar/ or exp saudi arabia/ or exp syria/ or exp turkey/ or exp united arab emirates/ or exp yemen/ - exp arabs/ or exp jews/ - exp African Continental Ancestry Group/ - exp Asian Continental Ancestry Group/ - exp Oceanic Ancestry Group/ |
| --- | --- | --- |

Table B: Initial PsycInfo subject headings and search strategy for cross-cultural articles on eating disorders

| - exp eating disorders/ - exp anorexia nervosa/ - exp binge eating disorder/ - exp bulimia/ - exp Body Image/ | and | - exp acculturation/ or exp cross cultural psychology/ or exp cultural sensitivity/ or exp multiculturalism/ - exp Cross Cultural Communication/ or exp Ethnography/ or exp Cross Cultural Psychology/ or exp Cross Cultural Differences/ or exp "Racial and Ethnic Differences"/ - exp "Culture (Anthropological)"/ or exp Ethnic Identity/ or exp Cross Cultural Treatment/ or exp Minority Groups/ - exp Ethnology/ - exp blacks/ - exp "Latinos/Latinas"/ or exp American Indians/ - exp asians/ or exp chinese cultural groups/ or exp japanese cultural groups/ or exp korean cultural groups/ or exp south asian cultural groups/ or exp southeast asian cultural groups/ or exp vietnamese cultural groups/ - minority groups/ or exp alaska natives/ or exp arabs/ or exp hawaii natives/ or exp indigenous populations/ or exp inuit/ or exp jews/ or exp pacific islanders/ or exp romanies/ - exp african cultural groups/ |
| --- | --- | --- |

Table C: Subject headings used for each ethnic group analysed

| Group | Medline subject headings | PsycInfo subject headings |
| --- | --- | --- |
| African | - exp africa/ - exp africa, northern/ - exp "africa south of the sahara"/ - exp African Americans/ - exp African Continental Ancestry Group/ | - exp Blacks/ - exp African Cultural Groups/ |
| Hispanic and Latin American | - exp hispanic americans/ - exp mexican americans/ - exp south america/ - exp argentina/ - exp bolivia/ - exp brazil/ - exp chile/ - exp colombia/ - exp ecuador/ - exp french guiana/ - exp guyana/ - exp paraguay/ - exp peru/ - exp suriname/ - exp uruguay/ - exp venezuela/ - exp central america/ - exp belize/ - exp costa rica/ - exp el salvador/ - exp guatemala/ - exp honduras/ - exp nicaragua/ - exp panama/ - exp latin america/ - exp Mexico/ | - exp *"Latinos/Latinas"/ |
| Middle Eastern | - exp middle east/ - exp afghanistan/ - exp bahrain/ - exp iran/ - exp iraq/ - exp israel/ - exp jordan/ - exp kuwait/ - exp lebanon/ - exp oman/ - exp qatar/ - exp saudi arabia/ - exp syria/ - exp turkey/ - exp united arab emirates/ - exp yemen/ - exp arabs/ - exp jews/ | - exp arabs/ - exp jews/ |
| Pacific Islands and Oceania^†^ | - exp melanesia/ - exp fiji/ - exp new caledonia/ - exp papua new guinea/ - exp vanuatu/ - exp micronesia/ - exp guam/ - exp palau/ - exp polynesia/ - exp hawaii/ - exp pitcairn island/ - exp samoa/ - exp tonga/ | - exp hawaii natives/ |
| East Asian and South-East Asian | - exp asia, southeastern/ - exp borneo/ - exp brunei/ - exp cambodia/ - east timor/ - exp Indonesia/ - exp laos/ - exp malaysia/ - exp mekong valley/ - exp myanmar/ - exp philippines/ - exp singapore/ - thailand/ - exp vietnam/ - exp far east/ - exp china/ - exp japan/ - exp korea/ - exp mongolia/ - exp taiwan/ | - exp chinese cultural groups/ - exp japanese cultural groups/ - exp korean cultural groups/ - exp southeast asian cultural groups/ - exp vietnamese cultural groups/ |
| South Asian* | - exp bangladesh/ - exp india/ - exp sikkim/ - exp pakistan/ - exp sri lanka/ | - exp South Asian Cultural Groups/ |
| South European | - exp greece/ - exp italy/ - exp mediterranean region/ - exp mediterranean islands/ - exp cyprus/ - exp malta/ - exp sicily/ - exp portugal/ - exp spain/ | - |
| East European | - exp europe, eastern/ - exp albania/ - exp baltic states/ - exp bosnia-herzegovina/ or exp bulgaria/ - exp "republic of belarus"/ or exp croatia/ - exp czech republic/ - exp hungary/ - exp "macedonia (republic)"/ - exp moldova/ - exp montenegro/ - exp poland/ - exp romania/ - exp russia/ - exp bashkiria/ - exp dagestan/ - exp moscow/ - exp siberia/ - exp serbia/ - exp slovakia/ - exp slovenia/ - exp ukraine/ - exp yugoslavia/ | - |

^†^ Citations on Australia or New Zealand only without a significant cross-cultural component were removed from this set.

* Citations referring to Native Americans or American Indians were removed from this set.

Table D: Limits applied for each publication type analysed

| Publication type | Medline limits | PsycInfo limits |
| --- | --- | --- |
| Reviews | “review articles” | “0800 Literature Review” or “0830 Systematic Review” [METHODOLOGY] |
| Case studies | Case Reports | “0200 clinical case study” or “1400 nonclinical case study” |
| Psychometric/validation studies | Validation studies | “2200 psychometrics & statistics & methodology” |
| Editorials | Editorial [PUBLICATION TYPES] | Editorial [DOCUMENT TYPE] |
| Comparative studies | Comparative study [PUBLICATION TYPES] | - |
| Randomized controlled trials | controlled clinical trial or randomized controlled trial | “2000 treatment outcome/randomized clinical trial” |
| Empirical studies | - | "0400 empirical study" |
